# Role of Cariprazine in Managing and Preventing Refractory Catatonia: A Case Study

**DOI:** 10.7759/cureus.70538

**Published:** 2024-09-30

**Authors:** Zana A Qasab

**Affiliations:** 1 Psychiatry, Shahid Hemn Mental Hospital, Sulaimanyah, IRQ

**Keywords:** bipolar disorder, cariprazine, ect, mood stabilizer, refractory catatonia

## Abstract

This case study explores the management of a 22-year-old male patient diagnosed with recurrent refractory catatonia, a neuropsychiatric condition characterized by motor, behavioral, and autonomic disturbances often associated with bipolar disorder. Despite comprehensive investigations, including normal results in tests such as complete blood count (CBC), liver function tests (LFT), thyroid function tests (TFT), renal function tests (RFT), C-reactive protein (CRP), creatine kinase (CKP), and serum electrolytes, the patient's condition persisted. Initial treatments with conventional therapies, such as benzodiazepines, proved unsuccessful. However, the introduction of cariprazine, an atypical antipsychotic, combined with electroconvulsive therapy (ECT), resulted in significant improvement. This case highlights the challenges of managing treatment-resistant catatonia and suggests cariprazine’s potential role in preventing catatonic relapses when other therapies fail. The patient’s sustained remission underscores the need for further investigation into cariprazine as a viable option for refractory cases.

## Introduction

Catatonia is a severe neuropsychiatric condition marked by motor, behavioral, and autonomic abnormalities. It can arise in the context of various psychiatric conditions, particularly mood disorders like bipolar disorder, as well as medical and neurological conditions [1]. The pathophysiology of catatonia is not fully understood, but it is believed to involve dysregulation in several neurotransmitter systems, particularly gamma-aminobutyric acid (GABA), dopamine, and glutamate [2]. This dysregulation may contribute to the characteristic symptoms of stupor, mutism, and motor immobility [3].

In the acute management of catatonia, benzodiazepines, especially the administration of lorazepam, can be initiated via sublingual, oral, or intramuscular routes, with a maximum dosage of 4 mg/day. The recommended starting dose is 2 mg, with an additional 2 mg administered if no therapeutic response is observed within three hours. If no clinical improvement is seen after one to two days of treatment, intravenous administration at higher doses, ranging from 6-16 mg/day, is considered the first-line treatment, offering rapid relief of symptoms in many patients [4]. Electroconvulsive therapy (ECT) is another established treatment, particularly for patients who do not respond to benzodiazepines or present with severe or life-threatening symptoms [5]. Despite these therapeutic interventions, certain cases of catatonia remain refractory, requiring innovative treatment approaches. The use of atypical antipsychotics, although primarily supported by anecdotal evidence, has shown promise in some instances. Low-dose risperidone, olanzapine, and clozapine have been reported as viable options, and emerging evidence suggests that cariprazine may also be an effective treatment choice for catatonia [6]. 

Cariprazine, a novel atypical antipsychotic, functions as a partial agonist at D3 and D2 dopamine receptors, exhibiting a higher affinity for D3 receptors. Cariprazine has received regulatory approval for the treatment of bipolar disorder. [7]. Its receptor selectivity, particularly its action on D3 receptors, suggests potential advantages in addressing it as a candidate for treating catatonia, particularly in cases where traditional treatments have failed [8]. This case study explores the use of cariprazine in combination with ECT to manage recurrent, treatment-resistant catatonia in a young male patient with bipolar disorder.

## Case presentation

Chief complaint: mutism and non-responsiveness to commands

A 22-year-old male presented to the psychiatric hospital in Sulaymaniyah, Iraq, with a five- to seven-day history of mutism and non-responsiveness. According to the family, he had not been eating or responding to commands during this period. During the consultation, the patient remained standing throughout the history-taking and examination, despite being offered a seat to sit. He exhibited no reaction to questions or instructions.

Mental state examination

The patient remained standing, did not move or blink, and avoided eye contact. He was mute, unresponsive to questions, and showed a blunt facial expression. Waxy flexibility was observed as his limbs retained the positions they were placed in by the examiner. The patient showed no reaction to verbal prompts or external stimuli, maintaining a catatonic, mute (mutism), and non-reactive state throughout the evaluation (negativism); also, he had posturing.

Psychiatric history

He had a previous diagnosis of bipolar disorder and was being treated by a psychiatrist. His previous medications included risperidone 4 mg and procyclidine 5 mg once daily, but he had discontinued this regimen several months before this presentation.

Medical and surgical history

The patient had no notable medical or surgical history.

Initial treatment plan

Given the severity of his condition, hospital admission was deemed necessary for further evaluation and management. Initial diagnostic investigations included a complete blood count (CBC), liver function tests (LFT), thyroid function tests (TFT), renal function tests (RFT), C-reactive protein (CRP), creatine kinase (CK), and serum electrolytes, all of which were within normal limits.

The patient was initially treated with lorazepam, titrated to 8 mg/day. After four days without any clinical improvement of catatonic features that he had, like waxy flexibility, mutism, negativism, and posturing, ECT was initiated. Six ECT sessions led to a significant improvement, and the patient was discharged with the following medications: olanzapine 5 mg once daily and procyclidine 5 mg once daily.

Recurrence and refractory catatonia

Ten days after discharge, the patient returned with a relapse of catatonic features: waxy flexibility, mutism, negativism, and posturing. He was readmitted, and a repeat workup was conducted, including brain magnetic resonance imaging (MRI), all of which yielded normal findings. The patient was again treated with lorazepam 8 mg/day, but no improvement was observed after four days. Consequently, eight ECT sessions (three times per week, one session every two days) were administered, with the catatonia resolving after the sixth session. To prevent relapse, a ninth ECT session was conducted as a prophylactic measure [9]. The patient was discharged on the following regimen: olanzapine 5 mg with fluoxetine 20 mg once daily at night, procyclidine 5 mg once daily, and lorazepam 2 mg for 10 days.

Second relapse and introduction of cariprazine

Despite the revised treatment plan, the patient experienced another relapse within seven to 10 days. Upon his third admission, the focus shifted to reevaluating potential organic causes, all of which were ruled out. The diagnosis of bipolar catatonia was reconfirmed.

At this stage, cariprazine, a novel antipsychotic with efficacy in managing bipolar disorder, was introduced. The patient was started on cariprazine 1.5 mg, titrated up to 4.5 mg in the evening, alongside lamotrigine with a titration schedule from 25 mg to 200 mg. Along with this, ECT was also employed as an adjunctive therapy, and after four sessions, the patient showed significant clinical improvement [10].

Follow-up and outcome 

The patient was discharged after the successful resolution of catatonia, with follow-up visits scheduled weekly for the first month, then monthly from the second to fourth month. During this period, the patient exhibited an euthymic mood and was able to resume his daily life activities, including starting a new job. There were no features of catatonia recurrence over four months.

## Discussion

This case highlights the challenges in managing refractory catatonia, particularly in the context of bipolar disorder [11]. While ECT and lorazepam are the mainstays of treatment, their limitations in preventing recurrence in some patients necessitate exploring alternative treatment strategies [12]. The introduction of cariprazine in this case played a pivotal role in both managing the acute catatonic episode and preventing further relapses. Cariprazine, a dopamine D3/D2 partial agonist with a higher affinity for D3 receptors, is effective in treating schizophrenia and bipolar disorder. Approved by the European Medicines Agency in 2017, it has demonstrated efficacy in managing both positive and negative symptoms of schizophrenia, preventing relapses, and treating manic, mixed episodes, and bipolar depression. Additionally, it shows promise in addressing refractory catatonia, particularly in bipolar disorder patients [13].

The combination of cariprazine, a dopamine D3/D2 partial agonist, with lamotrigine, a mood stabilizer used in bipolar disorder, and adjunctive ECT, appears to enhance treatment outcomes in refractory cases. This approach may provide significant benefits, particularly in patients unresponsive to traditional therapies like benzodiazepines or ECT alone, as evidenced by sustained remission in such cases [14].

This case highlights the importance of individualized treatment strategies for refractory catatonia, especially when standard treatments are ineffective. The unique mechanism of cariprazine, a dopamine D3/D2 partial agonist, combined with its demonstrated efficacy in this case suggests it may be a promising option for treatment-resistant catatonia. Cariprazine’s ability to target both manic and catatonic symptoms, alongside mood stabilizers like lamotrigine and adjunctive ECT, may enhance treatment outcomes [15]. Further research is necessary to explore the broader application of cariprazine in catatonia, particularly regarding optimal dosing, timing, and its integration with ECT and other therapeutic modalities.

## Conclusions

This case demonstrates the potential role of cariprazine in managing and preventing recurrent, refractory catatonia associated with bipolar disorder. The sustained remission achieved with cariprazine, combined with lamotrigine and ECT, suggests that this novel antipsychotic may offer a valuable therapeutic option for patients with treatment-resistant catatonia. Future studies are warranted to explore the broader applicability of cariprazine in similar cases, particularly in treatment-resistant populations.
